# Structural and biochemical evidence that ATP inhibits the cancer biomarker human aldehyde dehydrogenase 1A3

**DOI:** 10.1038/s42003-022-03311-1

**Published:** 2022-04-13

**Authors:** Albert Castellví, Raquel Pequerul, Vito Barracco, Judith Juanhuix, Xavier Parés, Jaume Farrés

**Affiliations:** 1 Department of Biochemistry and Molecular Biology, Faculty of Biosciences, Universitat Autònoma de Barcelona, 08193 Bellaterra, Cerdanyola del Vallès, Barcelona, Spain; 2 Alba Synchrotron, carrer de la Llum 2-26, 08290 Cerdanyola del Vallès, Barcelona, Spain

**Keywords:** Oxidoreductases, X-ray crystallography

## Abstract

Human aldehyde dehydrogenase (ALDH) participates in the oxidative stress response and retinoid metabolism, being involved in several diseases, including cancer, diabetes and obesity. The ALDH1A3 isoform has recently elicited wide interest because of its potential use as a cancer stem cell biomarker and drug target. We report high-resolution three-dimensional ALDH1A3 structures for the *apo*-enzyme, the NAD^+^ complex and a binary complex with ATP. Each subunit of the ALDH1A3-ATP complex contains one ATP molecule bound to the adenosine-binding pocket of the cofactor-binding site. The ATP complex also shows a molecule, putatively identified as a polyethylene glycol aldehyde, covalently bound to the active-site cysteine. This mimics the thioacyl-enzyme catalytic intermediate, which is trapped in a dead enzyme lacking an active cofactor. At physiological concentrations, ATP inhibits the dehydrogenase activity of ALDH1A3 and other isoforms, with a *K*_*i*_ value of 0.48 mM for ALDH1A3, showing a mixed inhibition type against NAD^+^. ATP also inhibits esterase activity in a concentration-dependent manner. The current ALDH1A3 structures at higher resolution will facilitate the rational design of potent and selective inhibitors. ATP binding to ALDH1A3 enables activity modulation by the energy status of the cell and metabolic reprogramming, which may be relevant in several disease conditions.

## Abstract (web summary)

Three X-ray structures are presented of human aldehyde dehydrogenase family 1 member A3 (ALDH1A3), a potential cancer stem cell biomarker (alone, with NAD+ and with ATP) and structure determination performed using molecular replacement.

## Introduction

The human aldehyde dehydrogenase (ALDH) superfamily comprises 19 enzyme isoforms catalyzing the NAD(P)-dependent irreversible oxidation of aldehydes to their corresponding carboxylic acids. ALDHs act on a wide variety of aldehyde substrates, some of them derived from lipids, such as retinoids, steroids and lipid peroxidation products. They play important roles in multiple biological processes, including oxidative stress response, lipid hormone metabolism, and cell differentiation. They also participate in oxidative phase I drug metabolism, contributing to chemotherapeutic drug resistance^1^. Recently, ALDHs have been involved in diabetes, obesity and metabolic reprogramming of tumor cells. Cytosolic ALDH1A enzymes, and specifically the ALDH1A3 isoform, are good markers of cancer stem cells and have been implicated in carcinogenesis, chemoresistance and relapse. The role of the increased ALDH activity in cancer stem cells is not well understood. Nevertheless, the stemness of this subpopulation of cells has been related to the participation of ALDHs in retinoic acid-mediated signaling pathways, and to the high antioxidant activity of these enzymes, specifically their ability to counteract the effect of reactive oxygen species. ALDH1A inhibition results in accumulation of toxic aldehydes with a concomitant increase in cell death and inhibition of tumor growth^2,3^.

More than 300 ALDH crystal structures are deposited in the Protein Data Bank, many of them corresponding to human ALDH1A1 and ALDH2. Most initial structure-function studies were performed with ALDH2 (in fact an ALDH1 family isoform, based on sequence identity) and residue numbering is usually referred to this isoform. ALDH1A1, ALDH1A2, ALDH1A3 and ALDH2 isoforms are tetrameric enzymes with 55-kDa subunits sharing 70% amino acid sequence identity. So far, only six ALDH1A3 crystal structures at a low resolution of 2.80–3.25 Å have been deposited, some in complex with inhibitors (Supplementary Table 1). These structures consistently show the ALDH monomers formed by three domains: NAD^+^-binding, catalytic and oligomerization domains. At the intersection of the substrate- and cofactor-binding pockets sits a strictly conserved cysteine residue (Cys302, ALDH2 numbering), which is essential for catalysis, acting as the active-site nucleophile. It is well accepted that the reaction starts with the nucleophilic attack of the aldehyde carbonyl group by the Cys thiol, and proceeds through the formation of a thiohemiacetal intermediate, hydride transfer to NAD^+^, deacylation and NADH release. The deacylation step requires the conserved Glu268, which acts as a general base to activate a water molecule that can make a nucleophilic attack on the thioacyl intermediate. This catalytic mechanism also explains the esterase activity of ALDH using the same active-site residues.

The cofactor-binding domain in ALDH deviates from the canonical Rossmann fold^4–7^, which consists of two βαβαβ motifs for dinucleotide binding, found in other NAD(P)^+^-dependent enzymes. In ALDH, the topological order of β strands and α helices is the same but the Rossmann fold contains five instead of the usual six β-strands and the mode of interaction of the cofactor is changed. In ALDH, the GXGXXG motif, usually found associated with the β1-αA loop in the canonical Rossmann fold, is now found to be in the β4-αD loop (as GXTXXG) and involved in interactions with the nicotinamide ring. In contrast to most other dehydrogenases, there are no positively charged residues surrounding the pyrophosphate moiety of NAD^+^, only the helical dipole provided by an α-helix. The adenosine ring rests in a cleft between two α-helices, while the phosphodiester bridge is solvent exposed making possible its rotational motion. This particular binding of the cofactor has functional consequences, as it allows for the nicotinamide ring to sample between different conformers, whereas the adenine portion remains relatively immobile. Two conformations, one extended (for hydride transfer) and one contracted (for hydrolysis) have been identified and are compatible with the two-step catalytic mechanism. The first conformation is associated with NAD^+^, as it brings the C4 atom of the nicotinamide at the right distance from the Cys302-Sγ, while the second conformation is suitable for NADH and permits Glu268 to act as a general base in the activation and orientation of the attacking water molecule. The conformational flexibility of the nicotinamide moiety of the cofactor is typically associated in crystal structures with increased thermal B-factor values. Thus, cofactor isomerization is needed for catalysis, differently from what is observed in other NAD(P)-dependent dehydrogenases, where changes in protein conformation and domain closure occur upon coenzyme binding and release^6^.

Mg^2+^ ions have been shown to stimulate the activity of ALDH1A3 and ALDH2 but impair the activity of ALDH1A1 and ALDH1A2^8^. This effect has been related to the different rate-limiting step for these isoforms: deacylation and cofactor dissociation, respectively. Mg^2+^ ions would induce a change in the relative population of the local nicotinamide conformation of the cofactor, by stabilizing one of the conformations of the phosphodiester region and thus the orientation of the nicotinamide ring relative to its ribose^6,7^.

Recently, we carried out the enzymatic characterization of the three human ALDH1A enzymes with non-retinoid substrates as well as retinaldehyde isomers^8^, and reported the inhibition kinetics with some novel compounds^9^. In view of the increasing importance of ALDH1A3 in various disease states and the need of structures at higher resolution for structure-based drug design, we focused here on contributing higher-resolution structures for ALDH1A3. In the current study, three previously unreported ALDH1A3 structures are presented: the *apo*-enzyme, the complex with NAD^+^, and with ATP bound to the adenosine-binding pocket of the cofactor-binding site. ATP inhibited ALDH activity at physiological concentrations, opening the possibility of ALDH modulation by the energy status of the cell.

## Results

### High-resolution structures of *apo* ALDH1A3 and of binary complexes with NAD^+^ and ATP

The three studied crystal forms of ALDH1A3, the *apo* form and the two complexes of the protein with NAD^+^ and ATP, were obtained under the same crystallization conditions. Only the additives in the crystallization solution were different in each case as described under Materials and methods. Data collection and model refinement statistics are shown in Table 1.

**Table 1:** Data collection and model refinement statistics.

PDB code	ALDH1A3 *apo*	ALDH1A3-NAD^+^ complex	ALDH1A3-ATP complex
	7QK7	7QK8	7QK9
**Data collection statistics:**			
Unit cell (*a*, *b*, *c*) (Å)	82.96, 90.13, 159.19	81.58, 89.52, 158.64	82.11, 90.01, 158.71
Space group	*P* 2_1_ 2 2_1_	*P* 2_1_ 2 2_1_	*P* 2_1_ 2 2_1_
Resolution range (Å)	48.44–2.29 (2.35–2.29)	48.00–1.89 (1.93–1.89)	48.19–1.78 (1.81–1.78)
Unique reflections	53,899 (4253)	92,123 (4461)	111,992 (4978)
Multiplicity	6.6 (6.1)	4.4 (4.5)	6.6 (6.1)
Completeness (%)	98.9 (95.7)	99.1 (98.6)	99.4 (90.9)
< I/σ_I_>	11.0 (1.7)	15.5 (1.9)	15.7 (0.9)
Wilson B-factor (Å^2^)	40.77	33.06	31.88
R-merge	0.124 (1.391)	0.051 (0.803)	0.062 (2.369)
Rmeas	0.134 (1.524)	0.057 (0.908)	0.067 (2.592)
Rpim	0.052 (0.613)	0.026 (0.417)	0.026 (1.034)
CC_1/2_	0.99 (0.73)	0.99 (0.74)	0.99 (0.57)
**Model refinement statistics:**			
R-work / R-free	0.1903 / 0.2270	0.1799 / 0.2039	0.1926 / 0.2150
Number of non-H atoms:			
macromolecules	7399	7400	7457
ligands	54	193	139
solvent	358	600	617
RMSD (bonds) (Å)	0.012	0.012	0.012
RMSD (angles) (°)	1.56	1.55	1.60
Ramachandran favored (%)	95.28	96.41	96.62
Ramachandran allowed (%)	4.41	3.08	3.28
Clashscore, all atoms	2.43	3.32	2.72
MolProbity score	1.57	1.51	1.50
Average B-factor (Å^2^):	56.55	40.91	45.39
macromolecules	56.27	39.94	44.48
cofactor	−	41.41	47.43
solvent	59.88	48.91	53.58

Values in parenthesis refer to the highest resolution shell.

**Fig. 1:**
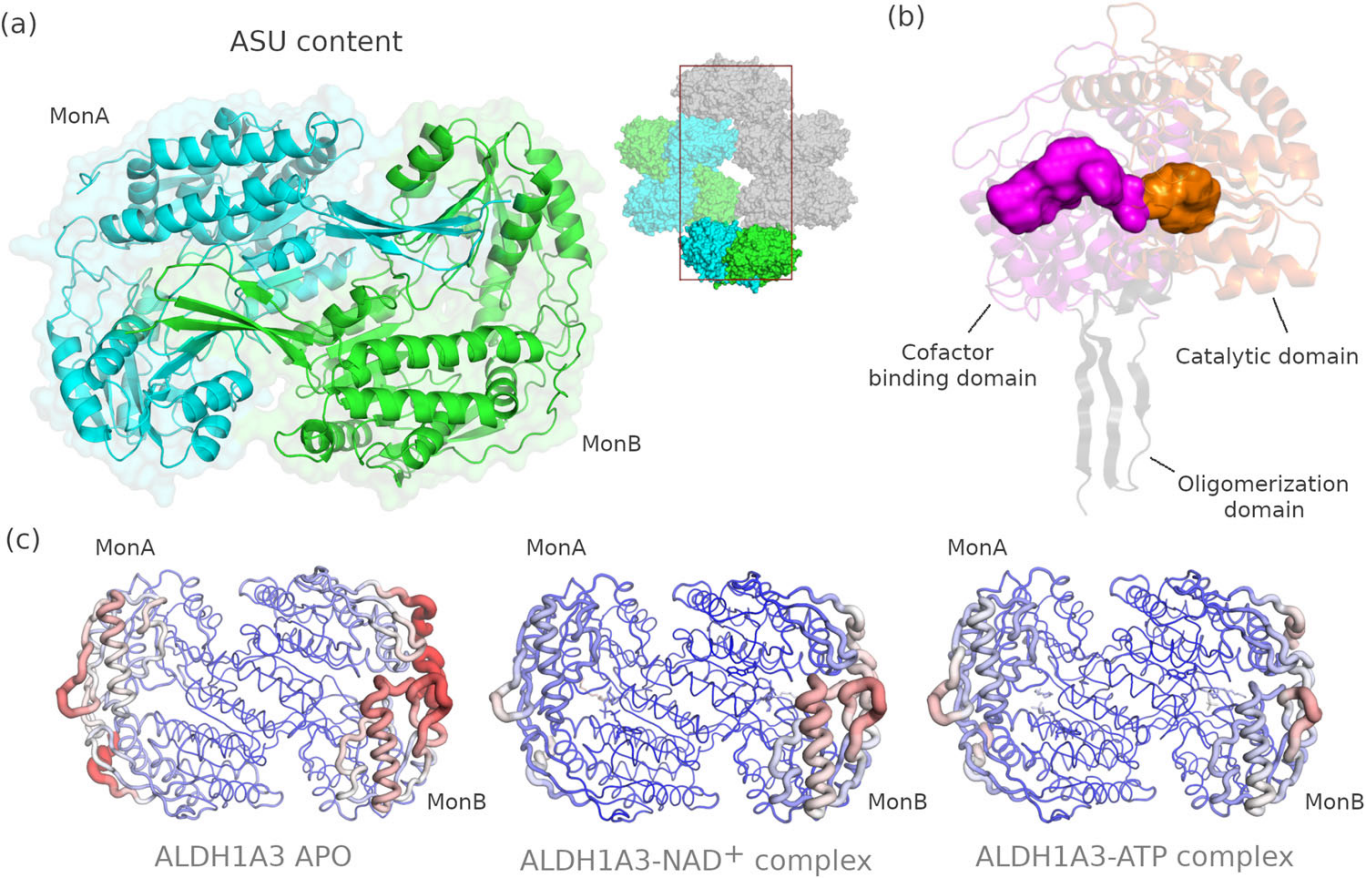
Structural description of ALDH1A3. **a** Half ALDH1A3 homotetramer contained in the asymmetric unit (left). The homotetrameric biological form appears in the crystallographic unit cell (right subfigure) after applying the symmetries of the space group. **b** Binding pockets in the cofactor (magenta) and catalytic (orange) domains. **c** Ribbon representation of the three described ALDH1A3 structures (PDB codes 7QK7, 7QK8 and 7QK9, respectively). The ribbon radius is scaled and normalized to the maximum B-factor reached in the *ALDH1A3 apo* structure. Residues in the main chain marked in red show the highest B-factor values, while those marked in blue show the lowest values.

The current structures show the highest resolution (1.78–2.29 Å) among all ALDH1A3 structures deposited in the PDB (Supplementary Table 1). All crystal structures contain a homodimer in the asymmetric unit (Fig. 1a, left). The relevantly biological assembly of the enzyme, the homotetrameric form, was confirmed by size-exclusion chromatography, and is obtained in the protein crystal when the space group symmetry is applied (Fig. 1a, right subfigure). As previously observed in other members of the enzyme superfamily, each ALDH1A3 subunit is composed of three structural domains: a catalytic domain, a cofactor-binding domain and an oligomerization domain (Fig. 1b). The volumes of the cofactor- and the substrate-binding pockets are very similar in all the structures, as calculated using the POVME algorithm. The volume of the pocket in the cofactor-binding domain is 444, 439 and 411 Å^3^ for the *apo* form, the ALDH1A3-NAD^+^ and the ALDH1A3-ATP complexes, whereas the volumes of the substrate-binding pockets are 231, 220 and 208 Å^3^ for the same forms, respectively (Fig. 1b). The cofactor- and catalytic-binding domains are defined by two sets of parallel beta sheets and alpha helices each, whereas the oligomerization domain is a set of three parallel beta sheets (Fig. 1b). The cofactor-binding domain contains a non-canonical Rossmann-fold motif, slightly different from that found in other NAD(P)^+^-dependent enzymes^6^. The three crystal structures described in this work do not present remarkable conformational changes among them and share the same disordered areas (Fig. 1c).

**Fig. 2:**
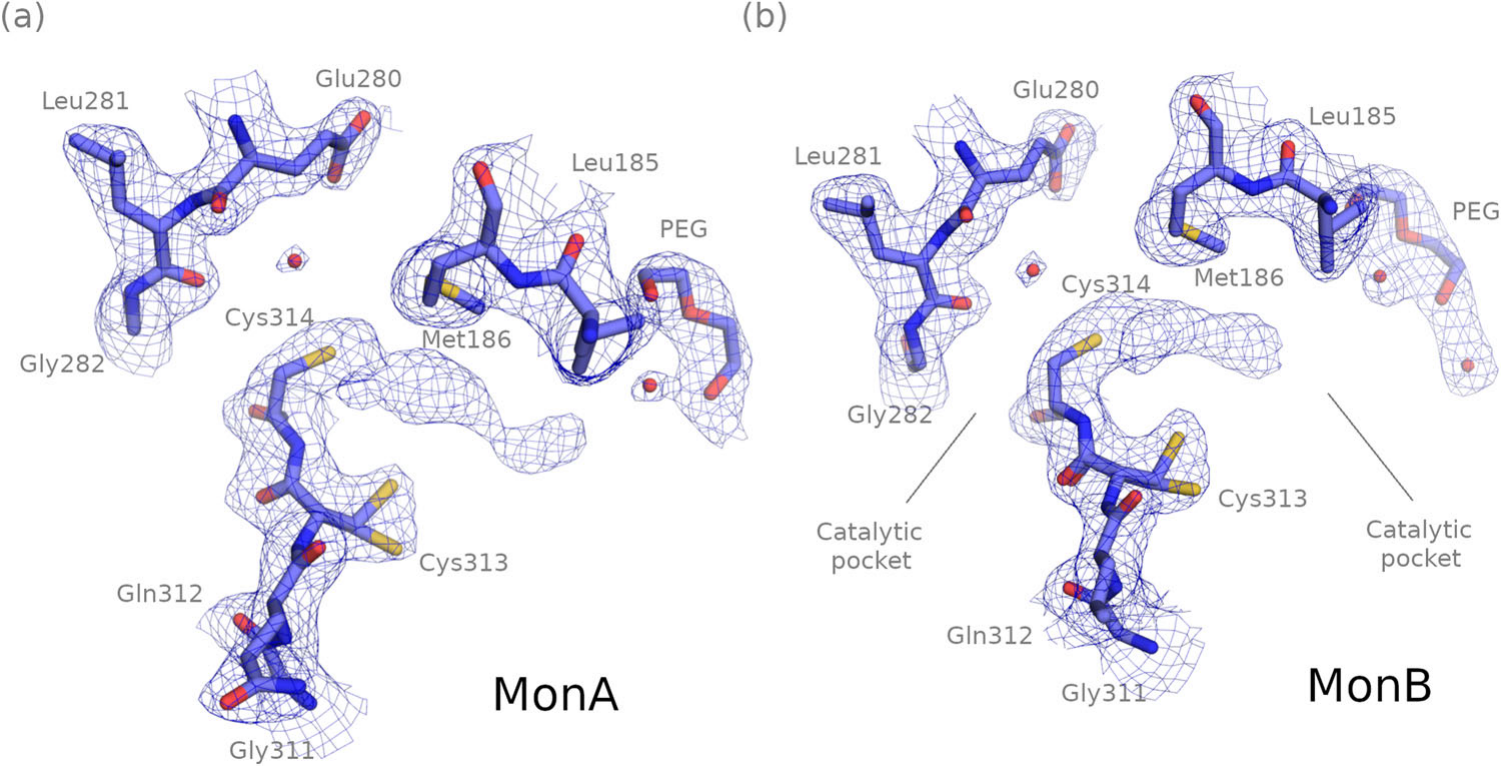
The *apo* ALDH1A3 crystal structure (PDB code 7QK7). 2Fo–Fc electron density map at σ = 1 and fitted crystal structure of the *apo* form of monomer A (**a**) and B (**b**) of ALDH1A3 close to the NAD^+^-binding and substrate-binding pockets.

The ALDH1A3 *apo* structure at 2.29-Å resolution shows a few electron density blobs inside the substrate-binding pocket near the catalytic Cys314 in both monomers (Fig. 2). These electron densities could not be modeled with any of the molecules present in the crystallization cocktail. A PEG molecule can also be seen within the substrate-binding pocket in both subunits of the crystal ALDH1A3 *apo* structure (Fig. 2). This PEG molecule remains conserved in the other structures obtained in this work.

**Fig. 3:**
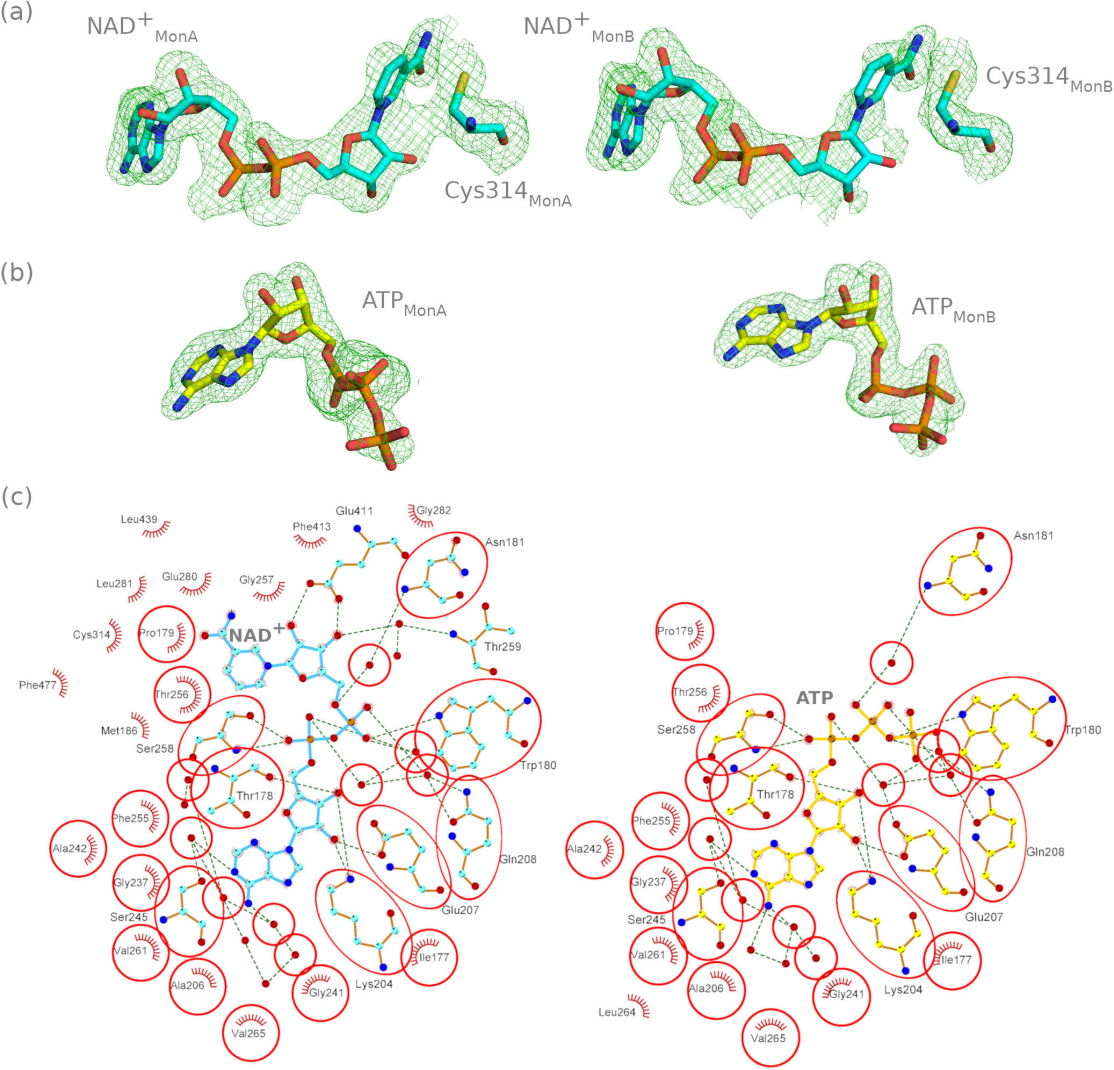
NAD^+^ and ATP interactions with ALDH1A3 (PDB codes 7QK8 and 7QK9, respectively). **a** Polder OMIT maps contoured at σ = 4 with fitted structures over the NAD^+^ molecules for each monomer of the ALDH1A3-NAD^+^ complex. **b** Polder OMIT maps contoured at σ = 6 around the ATP position in each monomer in the ALDH1A3-ATP complex, with the fitted ATP molecules. **c** LIGPLOT describing interactions between NAD^+^ and ALDH1A3 (left), and ATP and ALDH1A3 (right). Red circles show the structural elements placed in equivalent positions in ALDH1A3-NAD^+^ and ALDH1A3-ATP complexes.

Each monomer of the ALDH1A3-NAD^+^ complex at 1.89-Å resolution contains one NAD^+^ molecule positioned in each of the cofactor-binding pockets (Fig. 3a). As expected, the NAD^+^ molecule in both subunits is accommodated showing its extended conformation (suitable for hydride transfer) and interacting directly with residues Thr178, Trp180, Lys204, Glu207, Ser258, and Glu411 through hydrogen bonds, and to other 20 residues via hydrophobic interactions (Fig. 3c, left). The adenosine ribose is held in position by hydrogen bonds between the 2′ and 3′ hydroxyl oxygen atoms and Lys204. In addition, the 2′ hydroxyl oxygen forms a hydrogen bond with the sidechain of Glu207, which is part of the same loop. Thr178, forming a hydrogen bond with the 3’ hydroxyl oxygen through its backbone oxygen, is unique to ALDH1A3 in comparison to other ALDH1A and ALDH2 enzymes, which have an Ile residue at this position for this purpose. Only Glu411 makes two hydrogen bonds to the 2′ and 3′ hydroxyl oxygen atoms of the nicotinamide ribose. The position of the nicotinamide ribose is further stabilized by van der Waals contacts with the sidechain of Phe413. The extended conformation of NAD^+^ allows hydride transfer between the substrate and the C4 atom of the nicotinamide ring of the cofactor, a necessary step in the catalytic mechanism which would not be possible with the NAD^+^ molecule in contracted form. The catalytic Cys is modeled on both subunits as a single conformation pointing toward the substrate-binding pocket region (Fig. 3a). However, as suggested by the continuous electron density between Cys314 and the NAD^+^ molecule in monomer A, it is possible that the Cys residue was in a double conformation (Fig. 3a), as noticed in other ALDH structures, although its functional significance is uncertain^10–12^. This double conformation would give rise to some steric hindrances between the Cys and the cofactor and would explain the fractional occupancies of the latter molecule, having values equal to 1 in the adenosine moiety region but 0.74 and 0.64 in the nicotinamide part in monomers A and B, respectively.

The ALDH1A3-ATP structure at 1.78-Å resolution contains one ATP molecule in each subunit (Fig. 3b). The ATP molecule in monomers A and B is located in the adenosine-binding moiety of the cofactor-binding pocket and is accommodated via direct hydrogen bonding with Thr178, Trp180, Lys204, Glu207, and Ser258, and via hydrophobic interactions with ten additional residues (Fig. 3c, right). Interestingly, all the close contacts are exactly the same for the common part between NAD^+^ and ATP, including the hydrogen bonds, through water molecules, with Asn181, Gln208 and Ser245. Trp180 is hydrogen bound both to NAD^+^ and ATP pyrophosphate groups. The α- and β-phosphate groups of ATP have identical interactions to those of the NAD^+^ pyrophosphate, including four water molecules, while the γ-phosphate group is not showing any specific interaction. Eight hydrophobic interactions are also conserved. The highest B-factor and lower occupancy values in the NAD^+^ molecule are detected in the nicotinamide moiety in both subunits A and B, whereas the adenosine moiety displays the best ordered part of the whole molecule. The average B-factor and the occupancy values of the adenosine moiety in the NAD^+^ complex are very similar to those of the whole ATP molecule in the ALDH1A3-ATP complex. This illustrates the good anchoring of ATP in the adenosine-binding site of ALDH1A3. As anticipated, the average B-factor increased from α- to γ-phosphate groups of ATP (66–72 Å^2^), being highest for the latter, indicating more flexibility at this end of the ATP molecule (Supplementary Fig. 1).

**Fig. 4:**
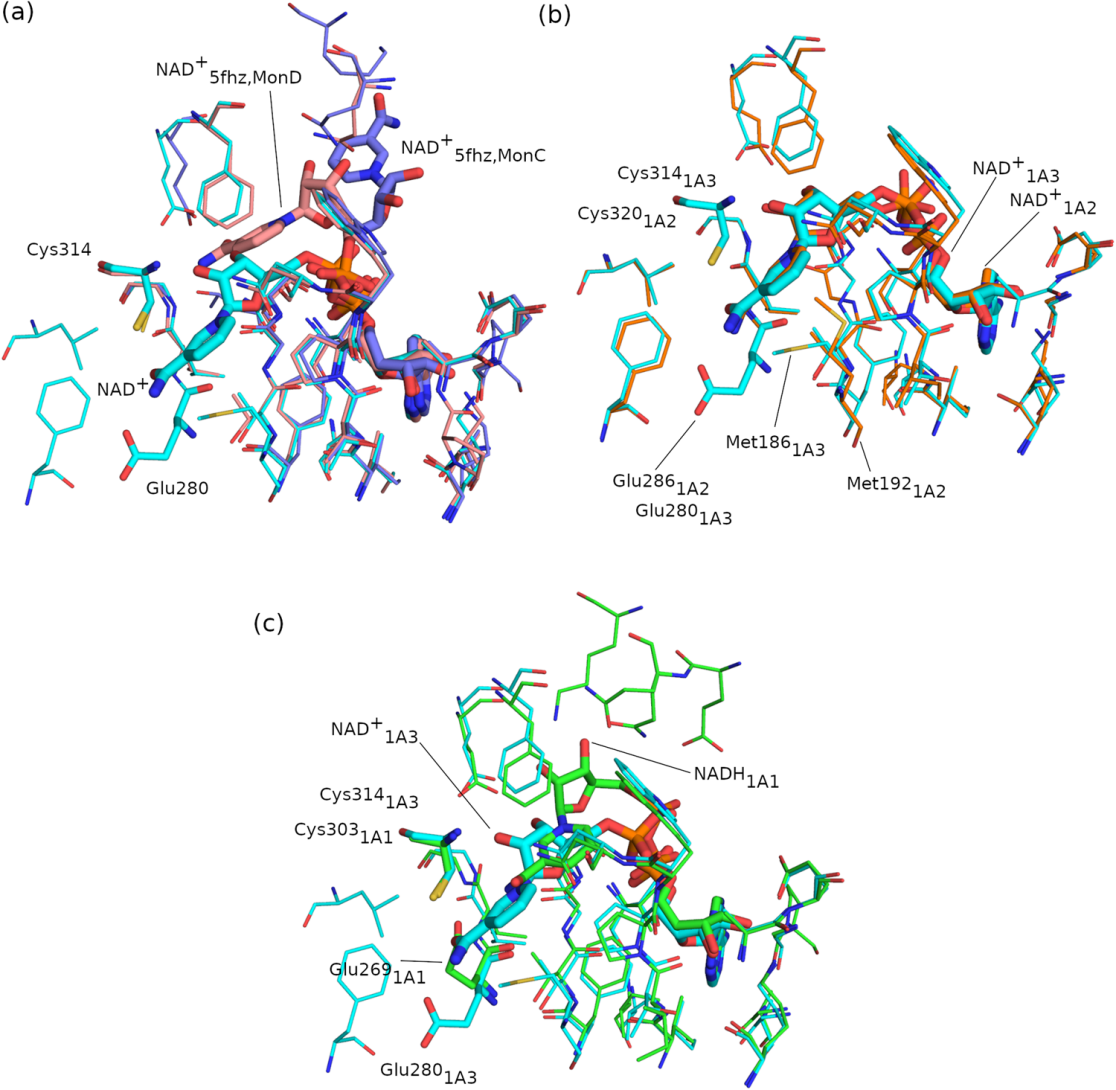
Structural alignment of present ALDH1A3-NAD^+^ structure with those of ALDH1A3, ALDH1A2, and ALDH1A1. **a** Alignment of current ALDH1A3-NAD^+^ (in cyan) with monomers C (in pink) and D (in purple) of the PDB 5FHZ structure (ALDH1A3 complexed with NAD^+^ and retinoic acid). **b** Alignment between ALDH1A3-NAD^+^ (in cyan) and PDB 6B5G structure (ALDH1A2 with NAD^+^ and 3-ethoxythiophen-2-yl){4-[4-nitro-3-(pyrrolidin-1-yl)phenyl]piperazin-1-yl}methanone, in orange). The conformation of all conserved residues remains unchanged except for the catalytic Cys320/Cys314 and the non-catalytic Met192/Met186 residues. **c** Alignment between the ALDH1A3-NAD^+^ (in cyan) complex and PDB 4WB9 structure (ALDH1A1 complexed with NADH, in green). Cofactor molecules and catalytic Cys and Glu residues are depicted with sticks while other residues interacting with the cofactor are shown with thin lines.

The ALDH1A3 structure complexed with NAD^+^ presented here has been compared to two previously published ALDH1A3 structures (Supplementary Table 1, PDB codes 5FHZ^13^ and 6S6W^14^). The low resolution (2.90 and 3.25 Å, respectively) of these two previously reported structures, which include a homotetramer in the asymmetric unit, did not show the canonical extended or contracted conformation for NAD^+^ (Fig. 4a). In fact, the nicotinamide ring of the modeled cofactors in monomers C and D of the homotetramer is pointing away from active-site Cys314. In contrast, the NAD^+^ molecules in the complex presented here show an extended conformation (suitable for hydride transfer), well accommodated in the cofactor-binding pocket, as seen in the highest resolution electron density maps (polder maps, Fig. 3a). Likewise, our ALDH1A3-NAD^+^ structure is consistent with all the ALDH-NAD^+^ structures having the cofactor in the extended form, as it is the case of the ALDH1A2 (PDB code 6B5G^15^, Fig. 4b). The superimposition between our ALDH1A3-NAD^+^ complex and the ALDH1A1-NADH complex (PDB code 4WB9^16^) evidences the movement of the cofactor to a contracted form (suitable for the hydrolysis step) and the catalytic Cys303/Cys314 and Glu280/Glu269 residues in ALDH1A1 (Fig. 4c).

**Fig. 5:**
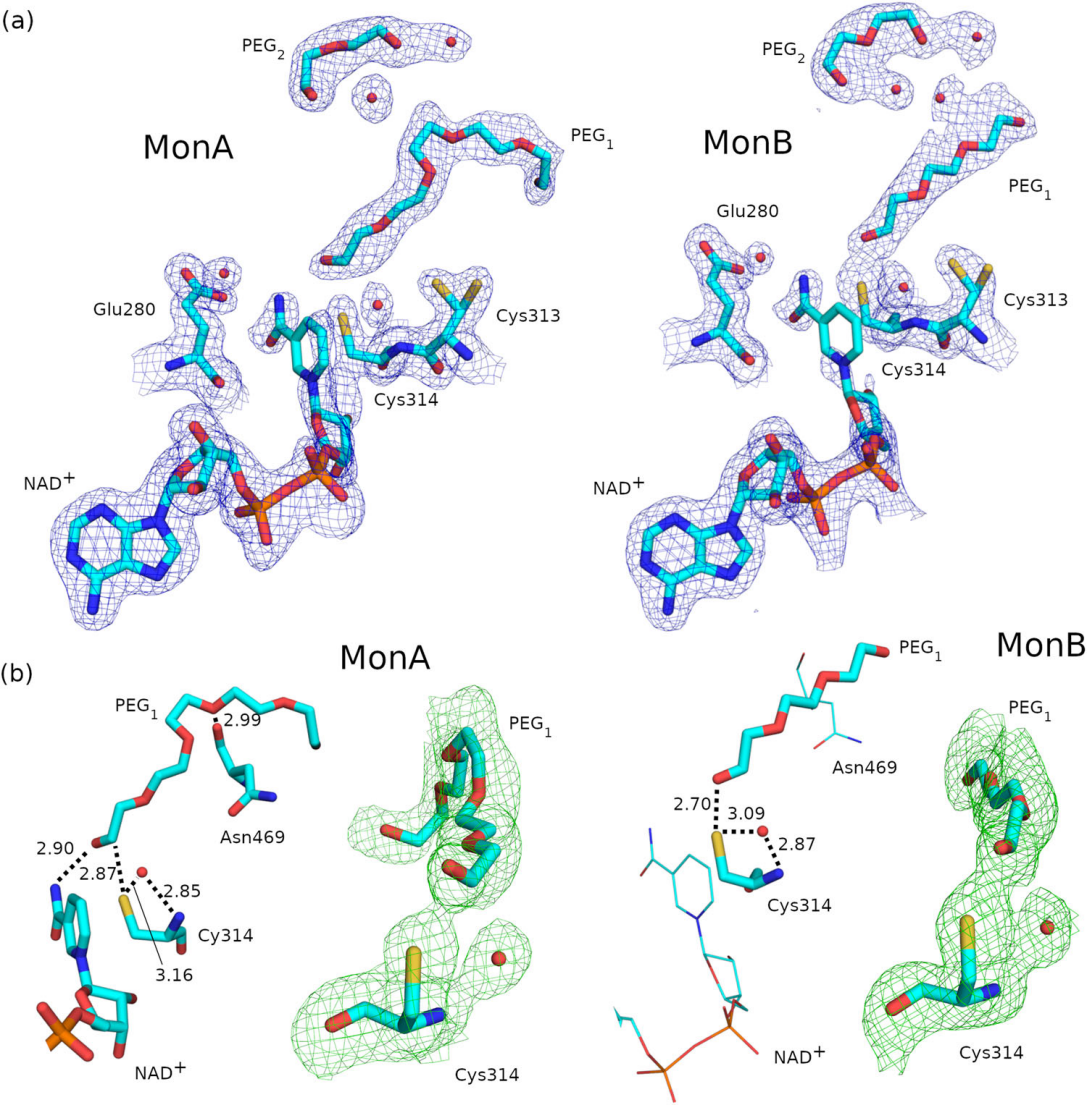
Cofactor and substrate structures in the ALDH1A3-NAD^+^ complex (PDB code 7QK8). **a** 2Fo–Fc electron density maps contoured at σ = 1 over Cys313, Cys314, Glu280, NAD^+^, PEG1, PEG2 and water molecules inside the cofactor and substrate-binding pockets. **b** For monomers A and B, hydrogen bonds of Cys314, Asn469 and NAD^+^ with PEG1, and polder OMIT maps at σ = 3.5 over Cys314 and PEG1. A highly conserved water molecule near Cys314 is depicted in red. The parts of the structure depicted with thin lines in monomer B represent those no longer interacting with the PEG1 molecule, with respect to monomer A.

The ALDH1A3-NAD^+^ complex contains a PEG molecule (PEG1) close to the Cys314 residue in the substrate-binding pocket, with 6 and 4 oxygen atoms in monomers A and B, respectively (Fig. 5a). Also, in the substrate-binding pocket there is a small PEG molecule (PEG2) in the same place in all subunits (Fig. 5a). None of the two PEG1 molecules were covalently bound to the Cys314 residues, as it can be deduced from the distances between the Cys314-S_γ_ and the PEG1 atoms and also from the electron density shown in Fig. 5b. Next to Cys314 there is a water molecule that, as it will be shown later, will play an important role in the position of the PEG1 molecule in the ALDH1A3-ATP complex (Fig. 5b). Finally, Cys313 appears in a double conformation in both monomers without forming a disulphide bond with Cys314 in any structure (Fig. 5a).

**Fig. 6:**
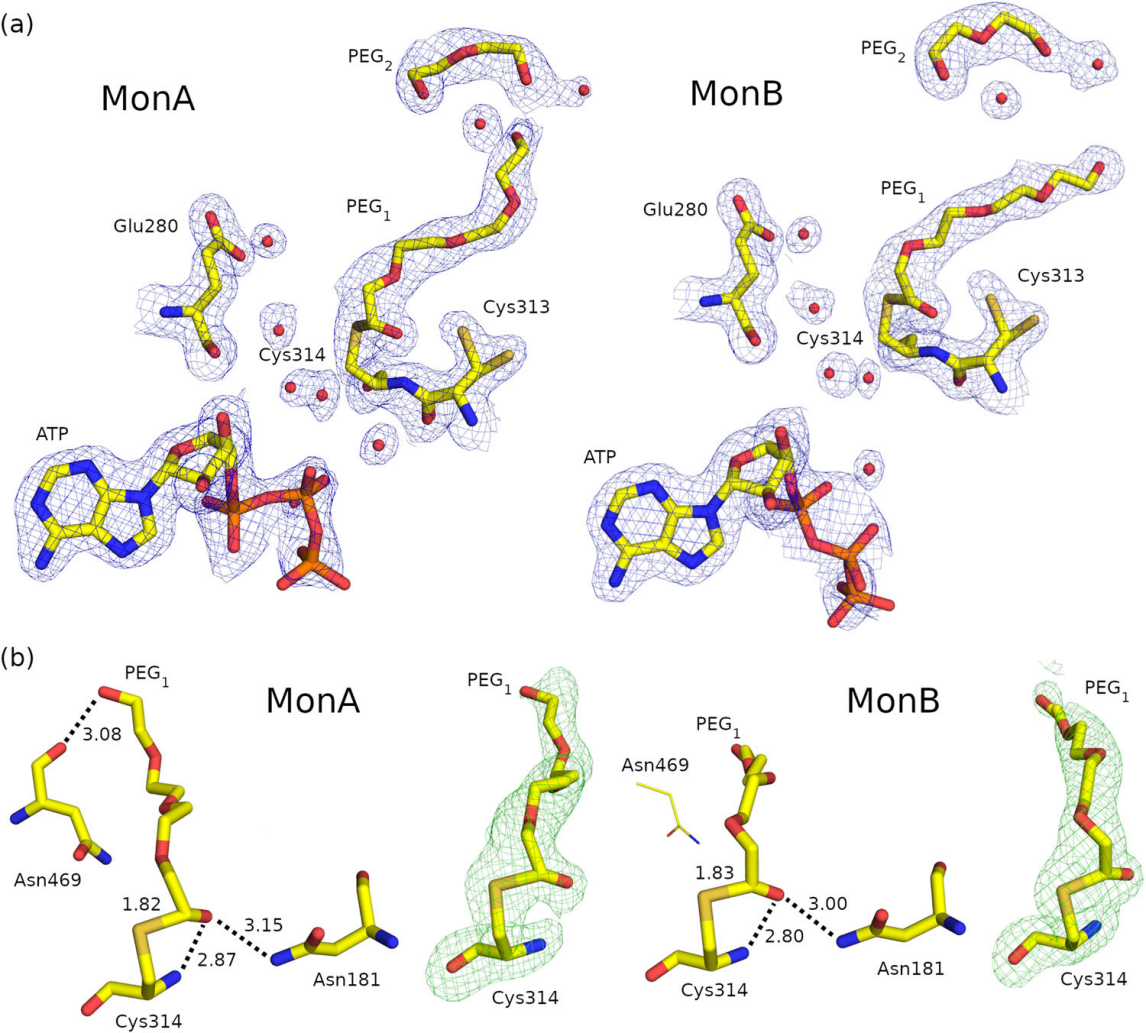
Cofactor and substrate structures in the ALDH1A3-ATP complex (PDB code 7QK9). **a** 2Fo−Fc electron density maps contoured at σ = 1 over Cys314, Cys313, Glu280, NAD^+^, PEG1, PEG2 and water molecules inside the cofactor and substrate-binding pockets. **b** Polder OMIT maps at σ = 5 over the Cys314 and PEG1, and crystallographic models showing hydrogen bonds between Cys314 and Asn181. The parts of the structure depicted with thin lines in monomer B represent those no longer interacting with the PEG1 molecule, with respect to monomer A.

The ALDH1A3-ATP structure also contains two PEG molecules (PEG1 and PEG2) inside the substrate-binding pocket in monomers A and B (Fig. 6a). However, in this case, the PEG1 molecule is covalently bound to Cy314 in both subunits with a distance between S_γ_-Cys314 and C_1_-PEG1 atoms of 1.82 and 1.83 Å for monomers A and B, respectively (Fig. 6b), which is a typical distance for a covalent C − S bond. In addition, the covalent union between Cys314 and the PEG1 molecule is observed in the continuous electron density shown in the polder maps. The O_1_-PEG1 atom is hydrogen bonded to the N-Cys314 and N_δ2_-Asn181 atoms in both monomers and occupies the position of the water molecule near Cys314 in the ALDH1A3-NAD^+^ structure (Fig. 6b). Additional water molecules are fitted in both monomers at the position of the nicotinamide ring of NAD^+^ in the ALDH1A3-NAD^+^ structure (Fig. 6a).

PEG molecules are frequently found as ligands in crystal structures and, particularly, in ALDH. PEG aldehyde formation has been described in solutions of PEG 400^17^ and its presence was previously reported in a human ALDH2 structure, where the authors confirmed the presence of aldehydes derived from PEG^6,18^, as well as in the tomato ALDH10 crystal structure (PDB code 4I9B^19^). As in the ALDH1A3-ATP complex described here, the C_1_-PEG-aldehyde atom in the ALDH10 structure is covalently bound to the S_γ_-Cys314 atom at a 1.86-Å distance (Supplementary Fig. 2). Therefore, we cannot rule out the possibility that PEG1 molecules covalently bound to Cys314 residues in the ALDH1A3-ATP complex could be PEG-aldehydes. The genuine interest of the ALDH1A3-ATP structure is that it can be interpreted as a true thioacyl-enzyme intermediate in the ALDH catalytic mechanism, which is trapped in a dead enzyme lacking an active cofactor. Although thioacyl-enzyme intermediates have been observed in other ternary complexes of the ALDH superfamily^19–21^, this is, along with human ALDH7A1, the only reported crystal structure of a human tetrameric ALDH showing the thioacyl-enzyme intermediate. Our results are in good agreement with the thiohemiacetal intermediate observed in tomato ALDH10 (PDB code 4I9B^19^, Supplementary Fig. 2) and in the non-phosphorylating glyceraldehyde 3-phosphate dehydrogenase from *Streptococcus mutants*^20^. The role of Asn181 in stabilizing the reaction intermediate through hydrogen bonding with the O atom of the carbonyl substrate, as suggested by our results, is consistent with similar bonds observed in other ALDH ternary complexes^19–21^.

### Inhibition of ALDH1A3 activity by ATP

The binding of ATP to a member of the ALDH superfamily is unprecedented in the literature. Since the ATP molecule is able to bind ALDH1A3 in the cofactor-binding pocket, the next step was to check if the ATP molecule could act as an enzyme inhibitor. A concentration of 2 mM ATP was chosen since it was that of the crystallization solution and is near its physiological concentration^22^. The inhibitory effect of ATP against several ALDH isoforms is shown in Table 2. ATP, at physiological concentrations, is inhibiting the dehydrogenase activity of ALDH, likely by interfering with the binding of cofactor. All ALDH isoforms tested were inhibited to various degrees. ALDH1A3 and ALDH3A1 were the most sensitive isoforms, while ALDH2 was affected the least. These results are consistent with the observed binding of ATP to the adenosine-binding site of ALDH1A3. The effect of ATP on ALDH enzymes is not dependent on the cofactor presence in the pre-incubation step with the reaction mixture. Thus, we observed the same effect by ATP when the reaction was started with NAD^+^ or hexanal as a substrate.

**Table 2:** Inhibition of ALDH activity in the presence of 2 mM ATP.

Remaining activity (%)				
ALDH1A1	ALDH1A2	ALDH1A3	ALDH2	ALDH3A1
43 ± 1	68 ± 2	22 ± 1	89 ± 1	25 ± 1

The inhibitory effect is expressed as the percentage of remaining activity (%) ± standard deviation, after 5-min pre-incubation in the absence of cofactor. *n* = 2, technical replicates for each isoform. Standard activity was measured at saturating concentrations of hexanal in the corresponding reaction buffer, containing 2 mM ATP and concentrations of the corresponding cofactor near *K*_*m*_ values.

**Fig. 7:**
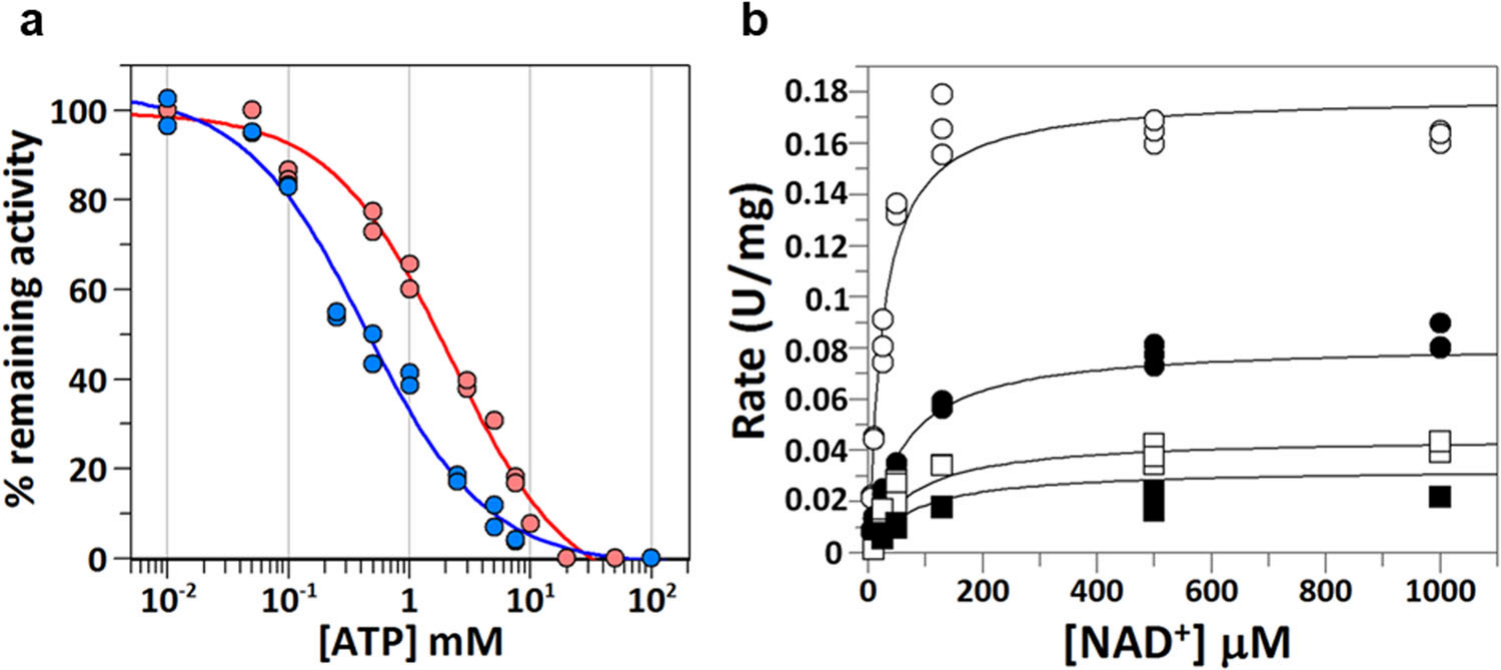
Inhibition kinetics of ATP against ALDH1A3. **a** Sigmoidal plots to determine IC_50_ values. The ALDH1A3 activity was assayed in 50 mM HEPES, 5 mM DTT, pH 8.0, without MgCl_2_ (blue curve) or including 30 mM MgCl_2_ (red curve). Hexanal was used as a substrate at saturating concentration (250 µM). NAD^+^ concentration was 24 µM in the absence of MgCl_2_, and 130 µM with 30 mM MgCl_2_. Experimental values with all replicate points are shown. *n* = 2, technical replicates for each ATP concentration (0.01–100 mM). **b** Michaelis-Menten plots fitted to the equation for mixed inhibition (Eq. 1). ATP concentrations (mM): 0.0 (empty circles), 2.0 (solid circles), 5.0 (empty squares) and 7.5 (solid squares). Hexanal was used as a substrate at a saturating concentration (250 µM). The values of kinetic parameters from this fit were: *V*_*ma*x_ = 0.18 ± 0.01 U/mg; *K*_*m*_ = 24 ± 2 µM; *K*_*i*_ = 0.48 ± 0.08 mM; *K*_*i*_*’* = 1.68 ± 0.12 mM. Experimental values with all replicate points are shown. *n* = 3, technical replicates for each NAD^+^ concentration (5–1000 µM). Parameter values are expressed as the calculated value ± standard error.

It is well known that the ATP-Mg complex, in the range of millimolar concentration, is the largest pool of Mg^2+^ ions within the cell. The complex follows a 1:1 stoichiometry, with a *K*_*d*_ value of 0.15 mM^23^. Since ALDH1A3 activity is activated by free Mg^2+^, it is conceivable that it also is finely modulated by the interplay between ATP and Mg^2+^. In order to check the effect of Mg^2+^ ions on the NAD^+^/ATP binding to ALDH1A3, the IC_50_ value for ATP was determined in the presence or absence of MgCl_2_ (Fig. 7a). The IC_50_ value of ALDH1A3 for ATP was 2.05 ± 0.23 mM, in the presence of 30 mM MgCl_2_ (under standard assay conditions), and 0.43 ± 0.06 mM, in absence of Mg^2+^ ions. In the latter case, a lower concentration of NAD^+^, near the *K*_*m*_ value (24 µM) was used because the lack of Mg^2+^ also affects NAD^+^ kinetics, lowering the *K*_*m*_ value for NAD^+^. For comparison, under standard assay conditions (130 µM NAD^+^), without adding Mg^2+^ ions, the IC_50_ value was 0.75 ± 0.05 mM. Overall, this supports the notion that Mg^2+^ ions activate ALDH1A3 also in the presence of ATP.

Thus, the effect of Mg^2+^ ions is to weaken ATP binding (IC_50_ value of 2.05 *versus* 0.43 mM). Since both ATP and NAD^+^ interact with Mg^2+^, the former effect could be interpreted as a result of either the destabilization of ATP binding or the strengthening of NAD^+^ binding. In order to unravel the confounding effect of Mg^2+^ on ATP in the presence of NAD^+^, the dissociation constant (*K*_*d*_) of ATP and ATP-Mg^2+^ could have been determined by a non-enzymatic method. Nonetheless, the fact that, in the absence of Mg^2+^, the *K*_*m*_ value for NAD^+^ decreases (from 130 to 24 µM) is consistent with the idea that Mg^2+^ is weakening NAD^+^ binding affinity. In that event, we could surmise that Mg^2+^ is also weakening ATP binding.

Then we determined the inhibition constant (*K*_*i*_) value and the type of inhibition for ATP against ALDH1A3, using various concentrations of NAD^+^, without adding Mg^2+^ ions. Data were plotted (Fig. 7b) and could be best fitted to the equation for mixed inhibition (Eq. 1), where *K*_*i*_ and *K*^*’*^_*i*_ are the constants for the competitive and uncompetitive parts of the inhibition:

1 $$v=\frac{\frac{{V}_{{\max }}\,{{\cdot }}\,[S]}{\left(1\;+\;\frac{[I]}{{K}_{i}{\prime} }\right)}}{{K}_{m}\,{{\cdot }}\, \frac{\left(1\;+\;\frac{\left[I\right]}{{K}_{i}}\right)}{\left(1\,+\;\frac{[I]}{{K}_{i}{\prime} }\right)}+[S]}$$

The *K*_*i*_ value is 0.48 ± 0.08 mM, which is similar to the IC_50_ value (0.43 ± 0.06 mM). The mixed inhibition type is not incompatible with ATP competing for binding to the same site as the adenosine moiety of the cofactor, as seen from the crystal structure. Various mechanisms could account for non-competitive inhibition patterns of inhibitors binding in the active site^24^. For instance, this behavior is feasible in enzymes that undergo multiple isomerization steps or bisubstrate kinetics following a compulsory ordered substrate addition or product release, as it is the case for ALDH. The rationale behind is that, while ATP and NAD^+^ bind to the same site, they may not always bind to the same exact enzyme species or conformational state.

We then investigated whether ADP was also an inhibitor of ALDH1A3. From inhibition kinetics (using hexanal or all-*trans*-retinaldehyde as a substrate), we observed that the ADP inhibitory effect was similar to that of ATP. This finding is in line with the small contribution of the γ-phosphate group to ATP binding. In contrast, AMP provided a much weaker inhibition if any (Table 3 and Supplementary Table 2).

**Table 3:** Inhibitory effect of ATP, ADP or AMP on the ALDH1A3 activity measured with hexanal.

[Compound] (mM)	ATP (%)	ADP (%)	AMP (%)
0.5	47 ± 5	56 ± 4	94 ± 2
5.0	9.3 ± 3.5	5.1 ± 1.1	79 ± 2

The inhibitory effect is expressed as the percentage of remaining ALDH1A3 activity (%) ± standard deviation, using 250 µM hexanal as a substrate after the addition of 0.5 and 5.0 mM ATP, ADP or AMP. *n* = 2, technical replicates. The enzyme and ATP/ADP/AMP mixtures were incubated for 5 min at room temperature, in the presence of 24 µM NAD^+^ cofactor, in 50 mM HEPES, 5 mM DTT, pH 8.0. The reaction was initiated by the addition of hexanal after pre-incubation. The effect of ATP and ADP was similar when 0.5 and 5.0 mM of each compound were added to the reaction. A much lower effect of AMP on ALDH1A3 activity was observed when these concentrations were used.

We also checked the inhibition of ALDH1A3 activity by ATP in the presence of the physiological substrate all-*trans*-retinaldehyde, using an HPLC-based methodology (Supplementary Fig. 3). We observed that the ALDH1A3 activity decreased from 63 to 16% when ATP concentration increased from 2.0 to 7.5 mM, confirming the inhibitory effect of ATP. The effect of ADP and AMP also mimicked what was found using hexanal as a substrate (Table 3 and Supplementary Table 2).

It is well established that ALDH isoforms possess esterase activity towards *p*-nitrophenyl esters, being the active-site Cys the essential nucleophile for both esterase and dehydrogenase reactions^25,26^. Thus, the hydrolysis of *p*-nitrophenyl acetate takes place at the same catalytic center which is responsible for the dehydrogenase activity^27^. Although NAD^+^ is not needed for esterase activity, it has been reported that it may enhance the acylation rate by promoting the attack of the active-site nucleophile on the carbonyl group of the substrate^28^.

Since ALDH1A3 also displays esterase activity in the absence of NAD^+^, we tested the effect of ATP against ALDH1A3 esterase activity. The *p*-nitrophenol production was followed in the presence or absence of NAD^+^, and combining with various concentrations of ATP (Supplementary Table 3). NAD^+^ enhanced esterase activity of ALDH1A3, which is consistent with deacylation being the rate-liming step^8^. The activity dropped down to 40% in the absence of cofactor. ATP could not substitute for NAD^+^ to enhance esterase activity, meaning that the adenosine moiety is not sufficient to activate the Cys nucleophilic attack. Remarkably, ATP alone inhibited esterase activity in a concentration-dependent manner. Likewise, when NAD^+^ was present, ATP also decreased esterase activity.

## Discussion

ATP binding sites are not uncommon in NAD(P)-dependent dehydrogenases and a few have been reported in members of the ALDH superfamily, but never before in human ALDHs. ATP-binding sites were described in the crystal structures of human mitochondrial NAD-dependent isocitrate dehydrogenase and NAD(P)-dependent malic enzyme, which are inhibited by ATP. A non-phosphorylating glyceraldehyde-3-phosphate dehydrogenase from *Thermoproteus tenax* participates in glycolysis and is regulated by ATP, and ALDH5A1 from *Arabidopsis thaliana* binds ATP at its NAD^+^-binding site^29–33^. Here, ATP functions as an active-site inhibitor of ALDH. The modulation of ALDH activity by ATP might have interesting physiological implications, since ALDH supplies the mitochondrial respiratory chain with NADH, which is the source of ATP. This could provide a regulatory mechanism for metabolic shifting to readjust the flux of ATP production.

The homeostatic level of intracellular ATP ranges from 0.5 to 5 mM^22^ and may change in tumor cells and upon chemotherapeutic treatment, meaning that ALDH1A3 activity could be physiologically modulated by changes in the energy status of the cell. It has been reported that intracellular ATP levels in cancer cells are higher than those in normal cells of the same tissue origin, and that drug-resistant cancer cells have even higher ATP levels than drug-sensitive cancer cells from which the resistant cells were derived^34,35^. ALDH activity, upregulated in some cancer types, may contribute to ATP production in cancer cells through the supply of NADH^36^. In this regard, ALDH1A3 expression has been linked to glucose consumption^37,38^. Recently, it has been shown that ALDH1A3 stimulates metastasis by altering glucose catabolism^39^ and that silencing of ALDH1A3 leads to significantly decreased glucose consumption, reduced lactate and ATP production in cancer cells^40^. There is some evidence that ALDH1A3 promotes glycolysis through the activation of the PI3K/AKT/mTOR signaling pathway^39^. On the other hand, it is conceivable that cellular ATP levels may modulate ALDH1A3 activity by a negative feedback mechanism, similarly to some enzymes participating in the glycolytic pathway, which may be relevant in other disease states such as obesity and diabetes. Overall, ALDH1A3 and possibly other ALDH isoforms are emerging as targets of choice for the control of cell proliferation and metabolic disorders.

In summary, three high-resolution structures have been elucidated for ALDH1A3, one of them revealing a previously unreported ATP-binding site in the adenosine-binding moiety of the cofactor-binding site. Enzymatic assays revealed that ATP is a good inhibitor of both the dehydrogenase and esterase activities of ALDH1A3. In fact, ATP at physiological levels effectively modulates ALDH1A3 activity. This allows us to speculate that the energy status of the cell is associated with ALDH1A3 and its potential role in metabolic reprogramming in disease conditions, such as cancer, diabetes and obesity. The availability of high-resolution 3D structures paves the way for structure-based inhibitor design and discovery of drugs targeting ALDH1A3.

## Methods

### ALDH isoform purification and ALDH1A3 crystallization

Each of ALDH1A1, ALDH1A2, ALDH1A3, ALDH2 and ALDH3A1 cDNA was cloned into the pET-30 Xa/LIC expression vector. The expression of the His_6_-tagged protein in *E. coli* strain BL21(DE3) pLysS was induced at 20 °C by 1 mM IPTG at OD_600_ 0.8–1 and incubated overnight in 1 L 2xYT culture media. Cells were subsequently pelleted by centrifugation at 4 °C and then resuspended in 30 mL of lysis buffer (20 mM Tris-HCl pH 8.0, 500 mM NaCl, 10 mM DTT and 20 mM imidazole). The resuspended cells were disrupted by sonication and the pellet was discarded. The supernatant was loaded in 1 mL nickel ion affinity chromatography column previously equilibrated with 20 mM Tris-HCl, pH 8.0, 500 mM NaCl and 20 mM imidazole. Highly pure fractions of each ALDH isoform were eluted with 200 mM imidazole and buffer was changed to 5 mM Tris-HCl, pH 8.0, 150 mM NaCl and 10 mM DTT.

ALDH1A3 crystals were grown by using the hanging drop method. Five µL of protein solution were mixed with 5 µL of crystallization solution and the resulting drop was equilibrated for a week against a reservoir containing 500 µL of crystallization solution at room temperature. Protein solution consisted of 5–10 mg/mL of ALDH1A3 in storage buffer supplemented with 10 mM MnCl_2_ for the *apo* form, 2 mM NAD^+^ for the NAD^+^ complex and 2 mM ATP for the ATP complex. The crystallization solution consisted of 100 mM BIS-Tris, pH 5.5, 2–2.2 M ammonium sulfate and 5% PEG 400. The seeding method allowed growing the crystals reproducibly reaching their maximum size (approximately 0.5 mm) in 3–4 days. The crystals were then transferred into a cryoprotecting solution consisting in 100 mM BIS-Tris, pH 5.5, 2.5 M ammonium sulfate, 5% PEG 400 and 15% glycerol prior to be loop-mounted and stored in liquid nitrogen.

### X-ray data collection, data processing and structure refinement

Diffraction data collection at 100 K was performed in the BL13-XALOC beamline^41^ at the ALBA synchrotron using a Pilatus 6 M photon-counting detector (DECTRIS, Baden, Switzerland). The beam was set at 12.661 keV photon energy (λ = 0.979 Å). Each complete data set was indexed and integrated using XDS^42^ in the space group *P* 2_1_ 2 2_1_. Data sets were scaled and the structure factors were generated using AIMLESS^43^. Cell content analysis indicated two molecules in the asymmetric unit with a solvent content of 50.75%. The initial phases to solve the structure were calculated by molecular replacement with Phaser^44^ using the PDB entry 5FHZ as a model. Structure refinement was carried out with Buster^45^ and the examination of the electron density maps and manual rebuilding were performed with Coot^46^.

Polder maps were calculated with phenix.polder^47^. The POVME^48^ algorithm was used to measure the volume of the substrate and cofactor-binding pockets in all refined ALDH1A3 structures. LigPlot+^49^ was used to point the interactions between the enzyme and the solvent with the ligand. All figures in this work were performed with Pymol^50^.

### Fluorimetric assay for inhibition screening of ATP against ALDH1A isoforms, ALDH2 and ALDH3A1

Enzymatic activity was measured fluorimetrically using a Cary Eclipse (Varian) fluorimeter to follow the purification procedure and to check the enzyme concentration before each kinetic experiment^8^. Standard activity was measured at saturating concentrations of substrate using 30 µM hexanal for ALDH1A1, 250 µM hexanal for ALDH1A2, ALDH1A3 and ALDH2, and 750 µM hexanal for ALDH3A1. Regarding cofactor concentrations, all assays were performed at NAD^+^ concentrations near *K*_*m*_ values (25 µM NAD^+^ for ALDH1A1, 20 µM NAD^+^ for ALDH1A2, 130 µM NAD^+^ for ALDH1A3 and ALDH2, and 100 µM NADP^+^ for ALDH3A1)^8^. The inhibition screening was performed in 50 mM HEPES, 0.5 mM EDTA, 0.5 mM DTT, pH 8.0, for ALDH1A1, ALDH1A2 and ALDH2; 50 mM HEPES, 5 mM DTT, pH 8.0, for ALDH1A3; and 50 mM Tris/HCl, 5 mM DTT, pH 8.0, for ALDH3A1. ATP was dissolved in the appropriate reaction buffer, and the corresponding solution was added to the reaction mixture in a final concentration of 2 mM. ATP, hexanal as a substrate and enzyme were incubated for 5 min at 25 °C and the reaction was started by the addition of cofactor NAD^+^. The ATP effect was also tested when the 5-min pre-incubation was carried out in the presence of cofactor and the reaction was started by the addition of hexanal.

### Determination of kinetic constants and type of inhibition of ATP against ALDH1A3

The IC_50_ value of ALDH1A3 for ATP was determined using the experimental conditions described above, except for a concentration of cofactor near the *K*_*m*_ value in the absence of Mg^2+^ ions (24 µM NAD^+^). The effect of magnesium ions was also checked using a reaction buffer with MgCl_2_ (50 mM HEPES, 30 mM MgCl_2_, 5 mM DTT, pH 8.0). In this case, 130 µM NAD^+^ was used. The IC_50_ parameters were calculated by nonlinear regression analysis, fitting the initial rates to the appropriate equation using GraFit 5.0 (Erithacus Software). Parameter values were given as the calculated value ± standard error of ten different ATP concentrations (0–100 mM) run in duplicate, and the standard error value was less than 20% of the mean value. Activity assays to determine the type of inhibition and *K*_*i*_ value of ATP against ALDH1A3 were carried out in the absence of Mg^2+^ ions, using various concentrations of NAD^+^ cofactor at fixed substrate and inhibitor concentrations. Specifically, the concentration of hexanal was 250 µM and the concentrations of ATP were 2.0 mM, 5.0 mM and 7.5 mM, keeping the same conditions as those of the inhibition screening and IC_50_ experiments and using GraFit 5.0 for data analysis. The data obtained at different concentrations of ATP were fitted to the Michaelis-Menten equation to determine the values of kinetic parameters, *V*_*max*_ and *K*_*m*_. Then, results were fitted to the competitive, non-competitive, mixed and uncompetitive inhibition equations and the kinetic constant values were determined from the type of inhibition showing the most accurate fit. Parameter values were expressed as the calculated value ± standard error.

### Spectrophotometric assay to determine the effect of ATP on ALDH1A3 esterase activity

ALDH1A3 was assayed for esterase activity in a Cary Bio (Varian) spectrophotometer. Stock solutions of 10 mM *p*-nitrophenyl acetate were prepared with ethanol as a solvent to minimize the spontaneous hydrolysis, always ensuring that the final ethanol concentration in the assay did not exceed 1% (v/v)^51^. Reactions were performed at 25 °C in 50 mM HEPES, 0.5 mM DTT, pH 7.5, with 500 µM NAD^+^ as a cofactor, or in the absence of cofactor. In both cases, ATP was added at 500 µM (an equimolar concentration of cofactor) or 2 mM (IC_50_ concentration) in order to check the ATP effect in presence or absence of NAD^+^. The reactions were initiated by the addition of 50 µM *p*-nitrophenyl acetate to the enzyme solutions in 1-mL cuvettes. The formation of *p*-nitrophenol was followed at 400 nm (ε_400_ = 18,200 M^−1^ · cm^−1^)^52^ to monitor esterase activity, which was corrected for the non-enzymatic hydrolysis of *p*-nitrophenyl acetate.

### HPLC-based assay for the inhibitory effect of ATP on ALDH1A3 using all-*trans*-retinaldehyde as a substrate

An HPLC method was used to evaluate the retinaldehyde dehydrogenase activity of human ALDH1A3. Working solutions of retinoids were prepared by 10-min sonication in the presence of equimolar delipidated BSA^53^. The actual concentration of solubilized retinoid was determined based on the corresponding molar absorption coefficient (ε_370_ = 29,500 M^−1^·cm^−1^). The enzymatic reaction was carried out for 15 min at 37 °C in a final volume of 500 µL, after 5-min preincubation with 2 mM, 5 mM and 7.5 mM of each ATP, ADP or AMP, and using 50 mM HEPES, 5 mM DTT, pH 8.0, as the reaction buffer and a concentration of cofactor near the *K*_*m*_ value (24 µM NAD^+^). The reaction was stopped with 1 mL of cold hexane:dioxane:isopropanol (50:5:1, v/v/v), and retinoids were extracted by a two-step procedure with the same solvent mixture^54,55^. The aqueous phase was removed, and the organic phase was evaporated under a N_2_ stream. Retinoids were dissolved in 200 µL hexane and analyzed by a modification of a published method^56^, as follows. Retinoids were separated by HPLC on a Novapak^®^ Silica 4 µm, 3.9 × 150 mm in hexane:methyl-*tert*-butyl ether (96:4, v/v) mobile phase, at a flow rate of 2 mL/min, using a Waters Alliance 2695 HPLC. Elution was monitored at 370 nm and 350 nm for all-*trans*-retinaldehyde and all-*trans*-retinoic acid, respectively, using a Waters 2996 photodiode array detector. Quantification of retinoids was performed by interpolation of HPLC peak areas into a calibration curve of known retinoid concentration. All compound manipulations were performed under red light to prevent photoisomerization.

### Statistics and reproducibility

GraFit 5.0 (Erithacus Software) was used for kinetic data analysis. For most analyses, all technical replicates (*n* = 2 or 3) were shown, and parameter values were expressed as the calculated value  ±  standard error.

### Reporting summary

Further information on research design is available in the Nature Research Reporting Summary linked to this article.

## Supplementary information

Supplementary Information

Description of Additional Supplementary Files

Supplementary Data 1

Reporting Summary

## Data Availability

Structure deposition: The atomic coordinates and structural factors of the three human ALDH1A3 structures, i.e., the *apo* form, the ALDH1A3-NAD^+^ complex and the ALDH1A3-ATP complex, have been deposited in the Protein Data Bank (PDB, www.rcsb.org) with the accession codes 7QK7, 7QK8, and 7QK9, respectively. The source data underlying Fig. 7 are provided as Supplementary Data 1. Any remaining information related to the data generated or analyzed in this study is available from the corresponding author upon reasonable request.
