# Supplementary material for: Structural and biochemical evidence that ATP inhibits the cancer biomarker human aldehyde dehydrogenase 1A3
Source: Commun Biol. 2022 Apr 13;5:354. doi: 10.1038/s42003-022-03311-1 (PMC9007972; doi:10.1038/s42003-022-03311-1)
Supplement: Supplementary file 1 — Supplementary Information [file 42003_2022_3311_MOESM1_ESM.pdf]

Supplementary information

Structural and biochemical evidence that ATP inhibits the cancer biomarker human aldehyde dehydrogenase 1A3

Albert Castellví[1,2,3], Raquel Pequerul[1,3], Vito Barracco[1], Judith Juanhuix[2], Xavier Parés[1], Jaume Farrés[1,*]

[1]Department of Biochemistry and Molecular Biology, Faculty of Biosciences, Universitat Autònoma de Barcelona, 08193 Bellaterra, Barcelona, Spain.

[2]Alba Synchrotron, carrer de la Llum 2-26, 08290 Cerdanyola del Vallès, Barcelona, Spain.

[3]These authors contributed equally.

*To whom correspondence should be addressed (jaume.farres@uab.cat)

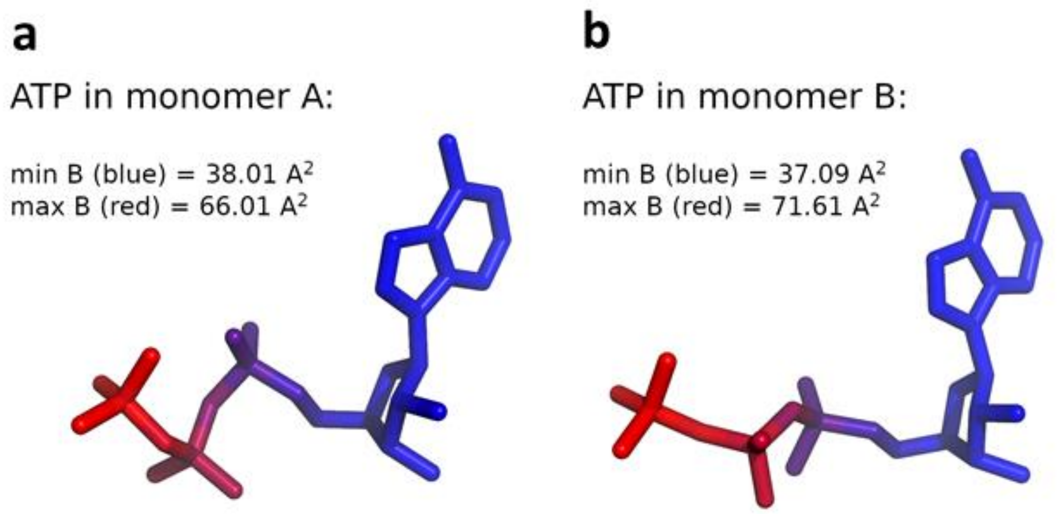

Supp. Fig. 1 | B-factor representation of the ATP molecule in the ALDH1A3-ATP complex. a Monomer A. **b** Monomer B. Maximum and minimum B-factor values are represented in red and blue, respectively.

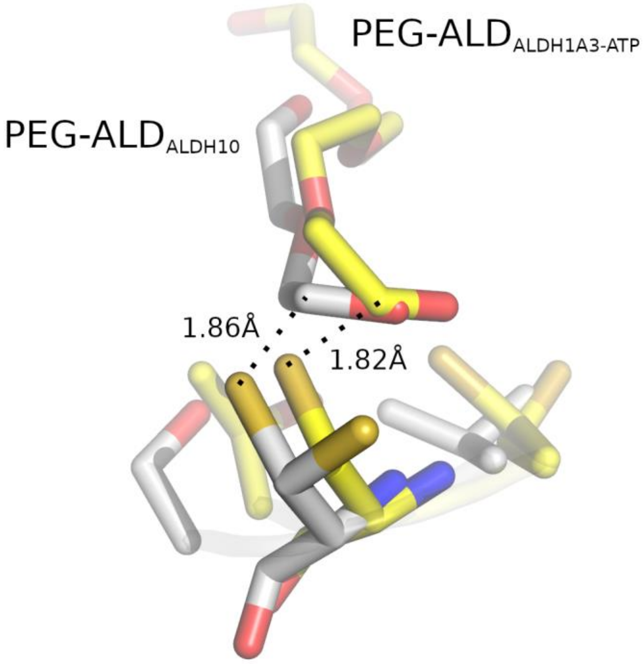

Supp. Fig. 2 | Superimposition of the crystal structure of thiohemiacetal intermediates in human ALDH1A3-ATP and tomato *(Solanum lycopersicum)* ALDH10. Superimposed Cys314 and Cys295 residues from the ALDH1A3-ATP complex and ALDH10 (PDB code 4I9B) structures show a very similar position of PEG1 and PEG-ALD molecules, respectively. Distances between each S_{γ}-Cys and C_1-PEG are very similar in the two structures.

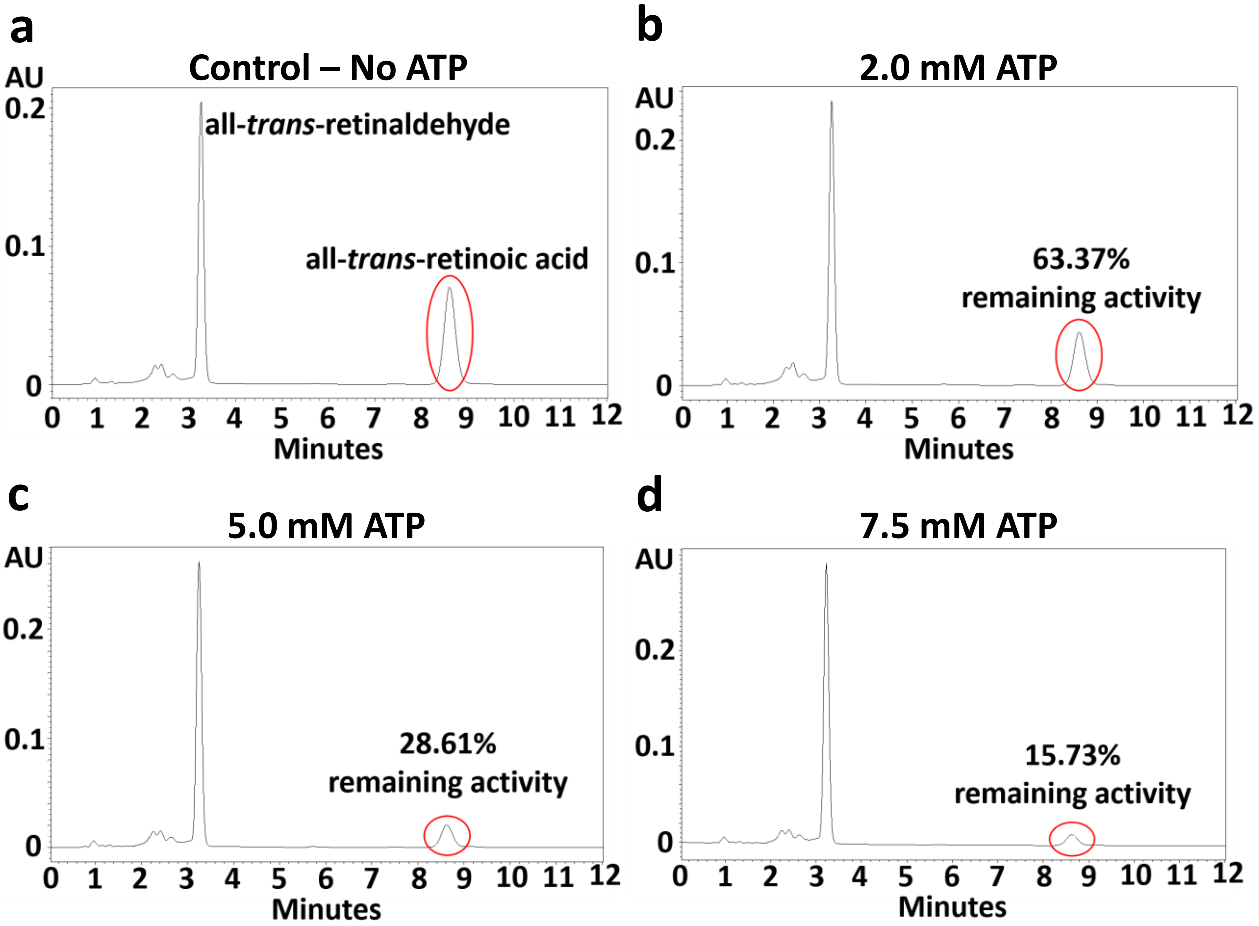

Supp. Fig. 3 | HPLC analysis of ALDH1A3 activity using all-*trans*-retinaldehyde as a substrate and ATP as an inhibitor. Elution profiles of all-*trans*-retinaldehyde (substrate) and all-*trans*-retinoic acid (product, indicated with a red circle) obtained after incubating in the presence of increasing concentrations of ATP. **a** Control without ATP (100% activity). **b** 2.0 mM ATP. **c** 5.0 mM ATP. **d** 7.5 mM ATP. ALDH1A3 showed 63.37, 28.61 and 15.73% of remaining activity with 2.0, 5.0 and 7.5 mM ATP, respectively.

Supplementary Table 1. Crystal structures of human ALDH1A3 deposited in the Protein Data Bank.

Cofactor	Other ligands	Resolution (Å)	PDB code	Released	Ref.
NAD^+	retinoic acid	2.90	5FHZ	Nov 2016	1
NAD^+	GA11	3.25	6S6W	Apr 2020	2
NAD^+	LQ43	3.25	6TE5	Nov 2020	2
NAD^+	MF13	2.90	6TRY	Jan 2021	3
NAD^+	NR6	2.95	7A6Q	Feb 2021	4
NAD^+	MCI-INI-3	2.80	6TGW	Jun 2021	5
–	–	2.29	7QK7	–	a
NAD^+	–	1.89	7QK8	–	a
–	ATP	1.78	7QK9	–	a

[a]Structures described in this work.

[1]Moretti A. *et al.* Crystal structure of human aldehyde dehydrogenase 1A3 complexed with NAD^+ and retinoic acid. *Sci. Rep.* **6**, 35710 (2016).

[2]Quattrini L. *et al.* Imidazo[1,2-a]pyridine Derivatives as Aldehyde Dehydrogenase Inhibitors: Novel Chemotypes to Target Glioblastoma Stem Cells. *J. Med. Chem.* **63**, 4603-4616 (2020).
GA11: 2,6-Diphenylimidazo[1,2-*a*]pyridine
LQ43: 6-(3,5-dimethoxyphenyl)-2-(4-methoxyphenyl)imidazo[1,2-*a*]pyridine

[3]Quattrini L. *et al.* Progress in the Field of Aldehyde Dehydrogenase Inhibitors: Novel Imidazo[1,2-a]pyridines against the 1A Family. *ACS Med. Chem. Lett.* **11**, 963-970 (2020).
MF13: 8-(4-Chlorophenyl)-2-phenyl-imidazo[1,2-*a*]pyridine

[4]Gelardi E.L.M. *et al.* A Selective Competitive Inhibitor of Aldehyde Dehydrogenase 1A3 Hinders Cancer Cell Growth, Invasiveness and Stemness In Vitro. *Cancers (Basel)* **13**, E356 (2021).
NR6: 3-(2-Phenylimidazo[1,2-*a*]pyridin-6-yl)benzonitrile

[5]Li J. *et al.* A specific inhibitor of ALDH1A3 regulates retinoic acid biosynthesis in glioma stem cells. *Commun Biol.* **4**, 1420 (2021).
MCI-INI-3: Methyl 5-(1,3-benzodioxol-5-yl)-2-phenyl-pyrazolo[1,5-*a*]pyrimidine-7-carboxylate

Supplementary Table 2. Inhibitory effect of ATP, ADP or AMP on the ALDH1A3 activity measured with all-*trans*-retinaldehyde.

[Compound] (mM)	ATP (%)	ADP (%)	AMP (%)
2.0	63.37	70.29	95.07
5.0	28.61	37.96	102.40
7.5	15.73	22.03	102.82

Percentage (%) of remaining ALDH1A3 activity, using 10 µM all-*trans*-retinaldehyde as a substrate after the addition of 2.0, 5.0 and 7.5 mM ATP, ADP or AMP. The enzyme and ATP/ADP/AMP mixtures were incubated for 5 min at room temperature, in the presence of 24 µM NAD^+ cofactor, in 50 mM HEPES, 5 mM DTT, pH 8.0. The reaction was initiated by the addition of all-*trans*-retinaldehyde substrate after the pre-incubation time. The effect of ATP and ADP was similar when 2.0, 5.0 and 7.5 mM of each compound were added to the reaction. No effect of AMP on ALDH1A3 activity was observed when these concentrations were used.

Supplementary Table 3. Effect of ATP on the ALDH1A3 esterase activity.

Parameter	0 µM NAD^+ 0 µM ATP	500 µM NAD^+ 0 µM ATP	0 µM NAD^+ 500 µM ATP	0 µM NAD^+ 2 mM ATP	500 µM NAD^+ 500 µM ATP	500 µM NAD^+ 2 mM ATP
nmols/min·mg	23	59	16	7	39	25
% remaining activity	40	100	27	11	67	42

Enzymatic activity of ALDH1A3 using 50 µM *p*-nitrophenyl acetate as a substrate in the presence and absence of NAD^+ or ATP, and combining various concentrations of ATP. The reaction was carried out in 50 mM HEPES, 0.5 mM DTT, pH 7.5, and the *p*-nitrophenol production was measured at 400 nm. Specific activity is shown in mU/mg and the remaining activity is expressed as the percentage relative to the activity measured with 500 µM NAD^+ in the absence of ATP.
