# Supplementary material for: Structural and biochemical evidence that ATP inhibits the cancer biomarker human aldehyde dehydrogenase 1A3
Source: Commun Biol. 2022 Apr 13;5:354. doi: 10.1038/s42003-022-03311-1 (PMC9007972; doi:10.1038/s42003-022-03311-1)
Supplement: Supplementary file 2 — Description of Additional Supplementary Files [file 42003_2022_3311_MOESM2_ESM.pdf]

Description of Additional Supplementary Files

File name: Supplementary Data 1
Description: Fig. 7 | Inhibition kinetics of ATP against ALDH1A3. a Sigmoidal plots to determine IC50 values. The ALDH1A3 activity was assayed in 50 mM HEPES, 5 mM DTT, pH 8.0, without $MgCl_2$ (blue curve) or including 30 mM $MgCl_2$ (red curve). Hexanal was used as a substrate at saturating concentration (250 µM). NAD^+ concentration was 24 µM in the absence of $MgCl_2$, and 130 µM with 30 mM $MgCl_2$. Experimental values with all replicate points are shown. n = 2, technical replicates for each ATP concentration (0.01-100 mM). b Michaelis-Menten plots fitted to the equation for mixed inhibition (Eq. 1). ATP concentrations (mM): 0.0 (empty circles), 2.0 (solid circles), 5.0 (empty squares) and 7.5 (solid squares). Hexanal was used as a substrate at a saturating concentration (250 µM). The values of kinetic parameters from this fit were: $V_{max} = 0.18 \pm 0.01$ U/mg; $K_m = 24 \pm 2$ µM; $K_i = 0.48 \pm 0.08$ mM; $K_i' = 1.68 \pm 0.12$ mM. Experimental values with all replicate points are shown. n = 3, technical replicates for each NAD^+ concentration (5-1000 µM). Parameter values are expressed as the calculated value ± standard error.
